# Can Echocardiography Aid in Diagnosing Vascular Rings?

**DOI:** 10.7759/cureus.50899

**Published:** 2023-12-21

**Authors:** Yasser A Bhat, Fahad Alhabshan, Abdulrahman Almesned, Abdullah Alqwaee

**Affiliations:** 1 Pediatric Cardiology, Prince Sultan Cardiac Center, Buraidah, SAU; 2 Cardiology, King Abdulaziz Medical City, Riyadh, SAU

**Keywords:** circumflex arch, double aortic arch, arch anomalies, echocardiography, vascular ring

## Abstract

Even though cardiac computed tomography and magnetic resonance imaging are the gold standard for evaluating the aortic arch in the context of vascular rings in children, echocardiography is usually the first-line modality. The echocardiographic evaluation of the aortic arch in the context of vascular rings in children has received little attention. This article details the step-by-step echocardiographic assessment of the aortic arch in vascular ring patients.

## Introduction and background

Vascular rings are a type of aortic arch abnormality that compress the trachea and esophagus [1]. A vascular ring may cause stridor, cough, dysphagia, wheezing, or dyspnea [2]. Catheter angiography and barium angiograms used in the past to diagnose vascular rings have been replaced by non-invasive modalities such as echocardiography, computed tomography (CT), and cardiac magnetic resonance imaging (cMRI) [3]. Currently, CT and cMRI are frequently used in preoperative planning; however, despite their diagnostic importance, CT and cMRI are associated with higher costs, radiation exposure (CT), and general anesthesia (cMRI). Transthoracic echocardiography (TTE) is usually the first tool to diagnose arch anomalies in the pediatric setting [3]. TTE offers many advantages in the evaluation of vascular rings. It is non-invasive, with no exposure to ionizing radiation. Moreover, it identifies a particular type of vascular ring and its anatomic characteristics and identifies associated intracardiac abnormalities [4]. The common variants of arch anomalies that may potentially produce a vascular ring are double aortic arch, right aortic arch (RAA) with aberrant left subclavian artery (ALSCA) and a left ductus arteriosus, left aortic arch with aberrant right subclavian artery and a right ductus arteriosus, RAA with mirror image branching pattern and a left-sided ductus arteriosus [2], and right or left circumflex aortic arch with ductus arteriosus on the opposite side of the arch [5].

Usually, a diverticulum of Kommerell gives rise to a ductus arteriosus opposite the side of the arch. Detailed assessment of vascular rings is essential for surgery, as the configuration of vascular rings may influence the type of surgical incision and cardiopulmonary bypass cannulation [3]. This article reviews TTE imaging techniques used in evaluating the aortic arch in the context of vascular rings. The precise diagnosis of a vascular ring requires a combination of all three views.

## Review

We propose three TTE views to define arch anatomy in the context of vascular rings, which, when combined, can lead to a precise diagnosis of vascular rings. (1) Suprasternal transverse view (SST) with probe position at 3 o'clock to visualize course and bifurcation of the first aortic arch branch. The first branch in the left aortic arch courses toward the right, and it courses toward the left if the arch is right-sided. (2) Suprasternal long-axis sweep (SSLA) with the probe position at 12 o'clock to identify tracheal cartilages, then titling the probe sideways to the left and right. As we tilt toward the left, the left aortic arch lies between the tracheal cartilages and the left pleura. In contrast, if the arch is right-sided while tilting to the right, the arch is visualized between the tracheal cartilages and the right pleura. (3) A high parasternal short-axis view (HPSA) with a posterior tilt shows the relationship between the descending thoracic aorta, the left and right lower pulmonary veins, and the vertebral body. The left descending aorta lies posterior to the left lower pulmonary vein (LLPV) on the vertebral body's left side. In comparison, the right descending aorta lies between the right lower pulmonary vein and the right side of the spine.

A combination of three views is required to diagnose a vascular ring accurately. The first view checks the arch-sidedness and aberrancy of subclavian arteries. The SSLA sweep determines the sidedness and rules out the double aortic arch, while the HPSA view rules out the circumflex aorta. The review's novelty lies in the combined use of these views, which may enable physicians to diagnose vascular rings accurately.

Left aortic arch with a normal branching pattern

**Figure 1:**
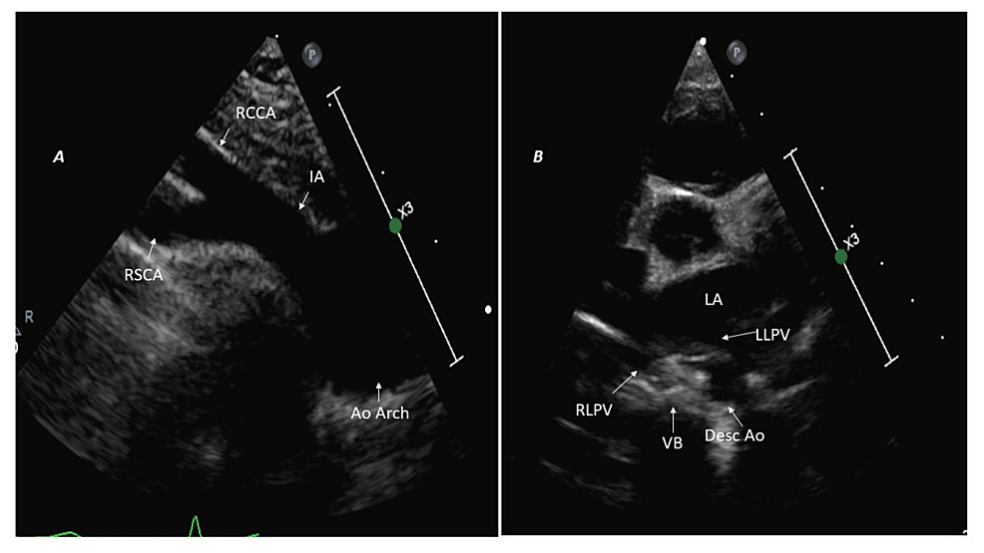
Left aortic arch with normal branching pattern and left-sided descending aorta (A) Suprasternal short-axis view: first branch (IA) courses toward the right and bifurcates. (B) High parasternal short-axis image: left-sided descending aorta (Desc Ao) located posterior to left lower pulmonary vein (LLPV) on the left side of the vertebral body (VB). Ao Arch: aortic arch; IA: innominate artery; RSCA: right subclavian artery; RCCA: right common carotid artery; RLPV: right lower pulmonary vein; LA: left atrium.

The normal left arch lies to the left of the trachea, crosses over the left main bronchus, and continues as the descending aorta to the left of the spine. The first branch passes to the right as the innominate artery gives rise to the right subclavian and right common carotid arteries, followed by the left common carotid and left subclavian arteries. The ductus arteriosus is on the left side, connecting the proximal left pulmonary artery to the proximal descending aorta, hence not forming a vascular ring [6]. In the SST, the first branch (right innominate artery) passes to the right and bifurcates into the right subclavian and common carotid arteries (Figure 1A). Moreover, the left descending thoracic aorta is seen posterior to the LLPV on the left side of the vertebral body in HPSA (Figure 1B).

In the SSLA, the left arch lies between the tracheal cartilage and the left pleura (Video 1).

**Video 1:** Left aortic arch Tracheal cartilages are visible when the probe is positioned at 12 o'clock in the suprasternal long-axis sweep. Tilting the probe leftwards reveals the left aortic arch and left pleura.

Left aortic arch with the aberrant right subclavian artery

**Figure 2:**
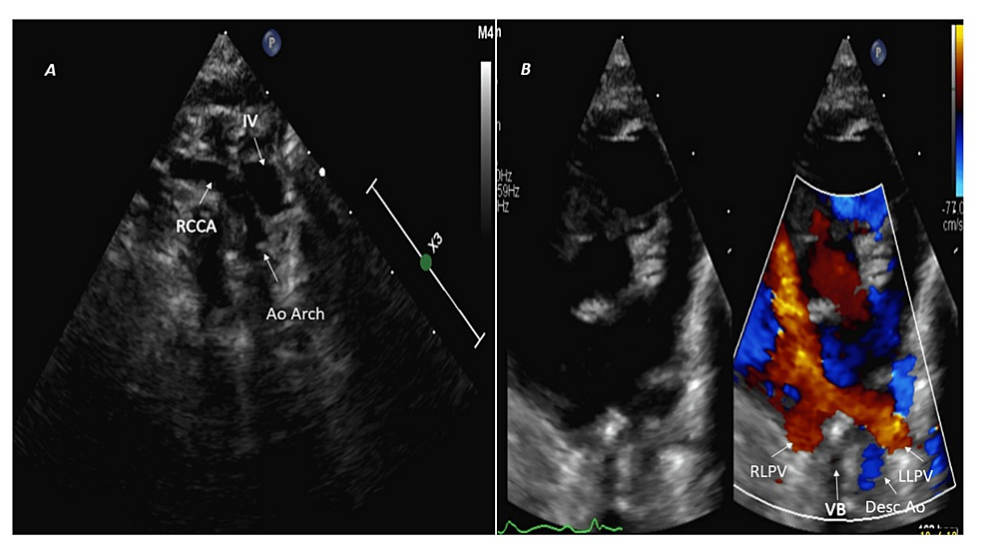
Left aortic arch with aberrant right subclavian artery (A) Suprasternal short-axis view shows the first branch (RCCA) courses toward the right but does not bifurcate. (B) High parasternal short-axis color comparing image: left-sided descending aorta (Desc Ao) located posterior to left lower pulmonary vein (LLPV) on the left side of the vertebral body (VB). Ao Arch: aortic arch; IV: innominate vein; RCCA: right common carotid artery; RLPV: right lower pulmonary vein.

In the SST view, the first branch courses to the right side; however, it does not bifurcate and continues as a single vessel, the right common carotid artery (Figure 2A). The aberrant right subclavian artery is the last aortic arch branch and usually runs a retroesophageal course [3]; therefore, it may be difficult to visualize on echocardiography except in infants. The left-sided arch between the trachea and left pleura can be imagined in the SSLA sweep (Video 1), and the left descending aorta is posterior to LLPV (HPSA view) (Figure 2B).

The Kommerell's diverticulum is the conical dilatation of the proximal part of the aberrant right subclavian artery near its origin from the aorta [7]. When the Kommerell's diverticulum is present, the ductus or ligamentum arteriosus is on the opposite side of the arch and arises from the junction between the Kommerell's diverticulum and aberrant right subclavian artery; therefore, it forms a vascular ring. In contrast, if Kommerell's diverticulum is absent, the ductus arteriosus is on the same side of the arch and does not form a vascular ring [3]. The diverticulum of the Kommerell coexists with the aberrant right subclavian artery in about 15% of cases [8] and may not be visualized on TTE as it lies posterior to the trachea and esophagus. The left aortic arch with the aberrant right subclavian artery is usually asymptomatic in children; however, it may cause dysphagia in 10% of adults [3,9].

Right aortic arch with mirror image branching pattern

**Figure 3:**
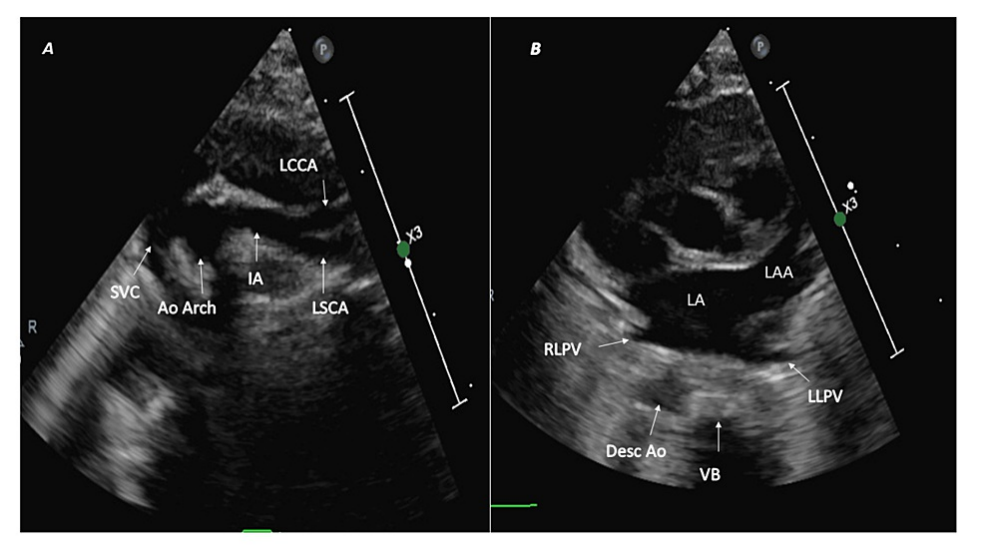
Right aortic arch with mirror branching pattern and right-sided descending aorta (A) Suprasternal short-axis view: first branch (IA) courses toward the left and bifurcates. (B) High parasternal short-axis image: right-sided descending aorta (Desc Ao) located posterior to right lower pulmonary vein (RLPV) on the right side of the vertebral body (VB). Ao Arch: aortic arch; IA: innominate artery; LSCA: left subclavian artery; LCCA: left common carotid artery; LLPV: left lower pulmonary vein; LA: left atrium; LAA: left atrial appendage.

The first branch is the left innominate, followed by the right common carotid and right subclavian, and the arch continues as the right descending aorta. The first branch courses toward the left (left innominate artery) and branches into the left subclavian and left common carotid arteries [7], as seen in the SST view (Figure 3A). The right arch lies between the tracheal cartilages and the right pleura (SSLA sweep, Video 2). Furthermore, the right descending aorta is located posterior to the right lower pulmonary vein and on the right side of the vertebral body (HPSA view) (Figure 3B).

**Video 2:** Right aortic arch Tracheal cartilages are visible when the probe is positioned in the suprasternal long-axis sweep at 12 o'clock. Tilting it rightwards reveals the right aortic arch and right pleura.

The ductus is typically anterior to the trachea, extending from the base of the left innominate artery to the left pulmonary artery; therefore, it does not encircle the trachea and esophagus to form a vascular ring [6]. The right aortic arch with mirrored branches is associated with congenital heart diseases like tetralogy of Fallot and truncus arteriosus in 98% of patients. Rarely, the ductus may extend between the right-sided descending aorta and left pulmonary artery and encircle the trachea and esophagus. In such cases, the intracardiac anatomy is typically normal; therefore, the vascular ring should be excluded in patients with a right aortic arch with mirror-image branching and normal intracardiac anatomy [10].

Right aortic arch with the aberrant left subclavian artery

**Figure 4:**
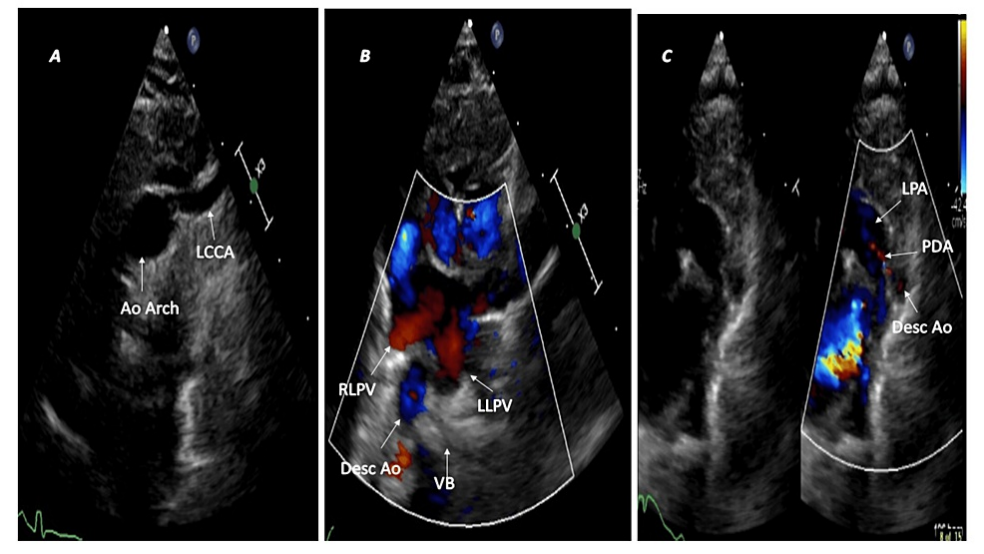
Right aortic arch with aberrant left subclavian artery and left-sided patent ductus arteriosus (A) The first branch (LCCA) courses leftwards but does not bifurcate. (B) The right descending aorta (Desc Ao) is posterior to the right lower pulmonary vein (RLPV) on the right side of the vertebral body (VB). (C) A tiny left-sided patent ductus arteriosus (PDA) completes the vascular ring. Ao Arch: aortic arch; LCCA: left common carotid artery; LLPV: left lower pulmonary vein; LPA: left pulmonary artery.

The first branch that runs on the left side but does not branch is the left common carotid artery (Figure 4A; SST view), followed by the right common carotid and right subclavian arteries. It is commonly associated with Kommerell's diverticulum (30-35%), which gives rise to the aberrant left subclavian artery. The right arch passes over the right mainstem bronchus and continues as the right descending thoracic aorta [11,12]. The right arch is visualized in the SSLA sweep (Video 2) and the right descending aorta in the HPSA view (Figure 4B). The left-sided ductus arteriosus completes the vascular ring (Figure 4C).

The first arch branch bifurcation in a right aortic arch may not be seen in the isolated left subclavian artery (SST view); however, this anomaly is rare and contributes to only 0.8% of the right arch anomalies [13]. In addition, isolation of the left subclavian artery is commonly associated with congenital heart defects, especially tetralogy of Fallot [3]. In contrast, the right aortic arch with the aberrant left subclavian artery is rarely associated with congenital heart disease. This abnormality is the second most common symptomatic vascular ring after a double aortic arch, and symptomatic patients typically present in childhood [12-14].

Circumflex aortic arch

**Figure 5:**
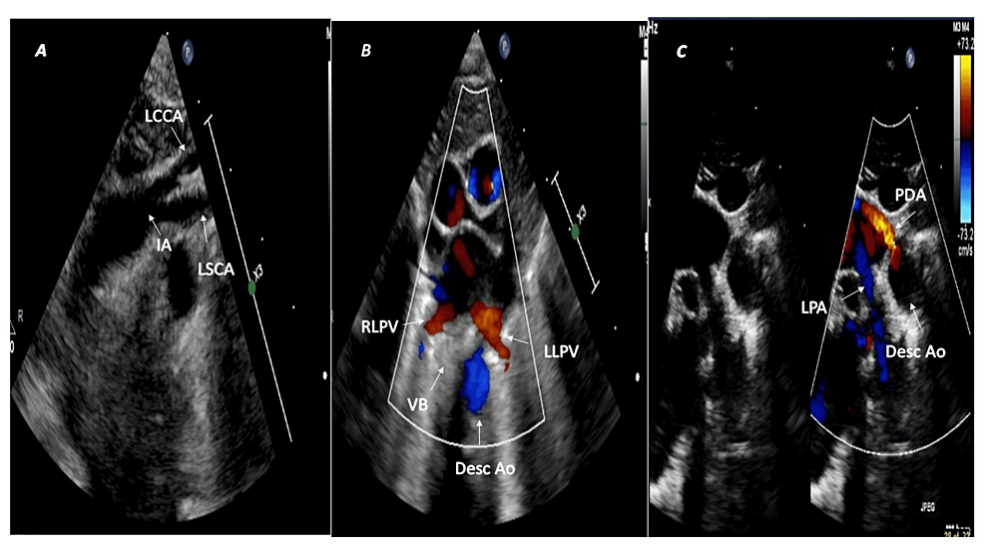
Right circumflex aorta and left patent ductus arteriosus (A) Right aortic arch with mirror image branching pattern. The left innominate artery (IA) courses leftwards and bifurcates into the left subclavian artery (LSCA) and left common carotid artery (LCCA). (B) The left descending thoracic aorta (Desc Ao) lies posterior to the left lower pulmonary vein (LLPV) on the left side of the vertebral body (VB). (C) The left patent ductus arteriosus (PDA) completes the vascular ring. RLPV: right lower pulmonary vein; LPA: left pulmonary artery.

In normal circumstances, the descending aorta persists on the same side as the aortic arch; however, in the circumflex aorta, the arch crosses the midline and passes behind the trachea and esophagus to the contralateral side, and a vascular ring is completed when a ligamentum arteriosus extends from the aortic arch to the pulmonary artery [3,5]. The left circumflex aorta has a left aortic arch, a right descending thoracic aorta, and a right ductus. The left transverse arch crosses the midline behind the trachea and esophagus and continues as the right descending aorta. The arch gives rise to the diverticulum of Kommerell, from which the right ligamentum arteriosus arises and connects to the right pulmonary artery, thereby completing the vascular ring [15]. In contrast, the right circumflex aorta has a right aortic arch that runs posteriorly behind the trachea and esophagus and descends as the left descending aorta. The vascular ring completes when the left ligamentum arteriosus arises from Kommerell's diverticulum and connects to the left pulmonary artery. The left subclavian artery may be aberrant in the right circumflex aorta [3,5]. Patients with circumflex aorta may become symptomatic in infancy or childhood due to tracheal compression, leading to severe respiratory compromise [3,15]. Echocardiography allows quick identification of the circumflex aorta (Figure 5).

Double aortic arch

**Figure 6:**
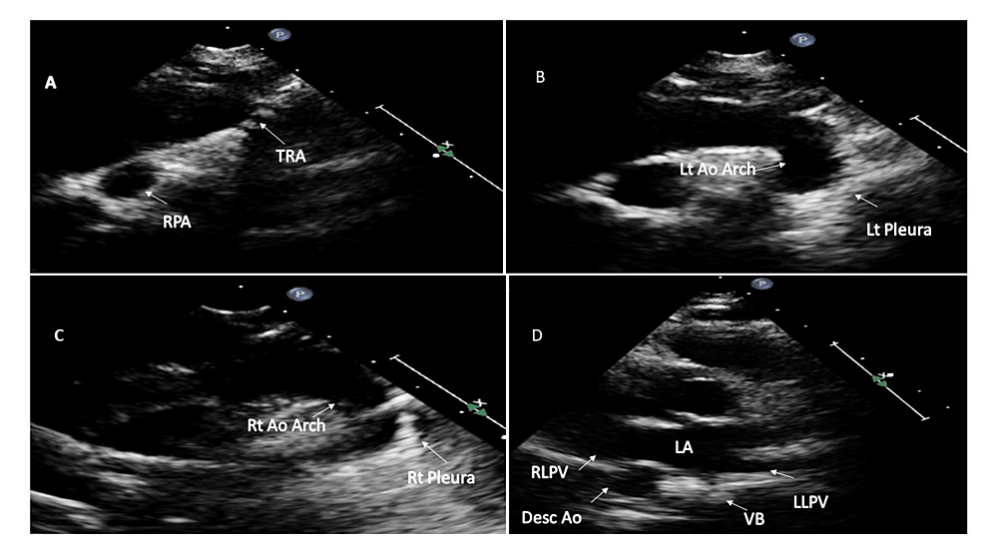
Sequential two-dimensional echocardiographic images of double aortic arch (A) Suprasternal long axis sweep with the probe position at 12 o'clock shows tracheal cartilages (TRA). (B) Tilting the probe leftwards shows the left aortic arch (Lt Ao Arch) and left pleura (Lt pleura). (C) Tilting the probe rightwards from 12 o'clock shows the right aortic arch (Rt Ao Arch) and right pleura (Rt pleura). (D) Right descending thoracic aorta (Desc Ao) in this patient with double aortic arch. RPA: right pulmonary artery; VB: vertebral body; RLPV: right lower pulmonary vein; LLPV: left lower pulmonary vein; RPA: right pulmonary artery; LA: left atrium.

In a double aortic arch, the ascending aorta splits into two arches that pass on either side of the trachea and esophagus, encircling them and then joining together to form a single descending aorta [16]. The right aortic arch is usually higher and larger than the left [3], and the descending aorta is usually contralateral to the dominant arch. Moreover, the common carotid and subclavian arteries arise separately from ipsilateral arches on both sides [16]. The most common arrangement is the larger right arch, the left descending aorta, and the left ligamentum arteriosus [3]. Echocardiography allows quick identification of two aortic arches in SSLA sweep (Figures 6A-6C and Video 3) and the sidedness of the descending thoracic aorta in the HPSA view (Figure 6D).

**Video 3:** Double aortic arch The color Doppler in the suprasternal long-axis sweep at 12 o'clock shows the tracheal cartilages. Tilting the probe leftward shows the left aortic arch and left pleura, while tilting it rightward from 12 o'clock shows the right aortic arch and right pleura. In this case, both the arches were patent and almost of equal size.

Patients with a double aortic arch become symptomatic in neonatal life [17], infancy, or childhood and are rarely associated with congenital heart defects [3].

## Conclusions

The echocardiography protocol described in the paper can allow for accurate diagnosis of vascular rings. Although cardiac CT and cMRI effectively determine the precise anatomy of arch malformation, accurately define its relationship to the surrounding structures, and provide a detailed evaluation of tracheoesophageal anatomy, TTE is a relatively easy way to identify vascular rings. Additionally, a proper echocardiographic evaluation will allow timely referral of patients with vascular rings to higher centers with cardiac CT and surgery facilities, especially in developing countries where expertise in cardiac CT concerning congenital heart disease is limited.
